# The DiaS trial: dialectical behavior therapy versus collaborative assessment and management of suicidality on self-harm in patients with a recent suicide attempt and borderline personality disorder traits - study protocol for a randomized controlled trial

**DOI:** 10.1186/1745-6215-15-194

**Published:** 2014-05-29

**Authors:** Kate Andreasson, Jesper Krogh, Bent Rosenbaum, Christian Gluud, David A Jobes, Merete Nordentoft

**Affiliations:** 1 Mental Health Centre Copenhagen, Faculty of Health Science, University of Copenhagen, Copenhagen, Denmark; 2 Mental Health Services, Copenhagen and Faculty of Health Sciences, University of Copenhagen, Copenhagen, Denmark; 3 Copenhagen Trial Unit, Centre for Clinical Intervention Research, Rigshopitalet, Copenhagen University Hospital, DK-2100 Copenhagen, Denmark; 4 Department of Psychology, The Catholic University of America, Washington, DC, USA; 5 Research Unit, Mental Health Centre, Bispebjerg bakke 23, Building 14, DK-2400 Copenhagen, NV, Denmark

**Keywords:** suicide prevention, deliberate self-harm, borderline personality disorder traits, BPD, dialectical behavior therapy, DBT, collaborative assessment and management of suicidality, CAMS

## Abstract

**Background:**

In Denmark 8,000 to 10,000 people will attempt suicide each year. The Centre of Excellence in Suicide Prevention in the Capital Region of Denmark is treating patients with suicidal behavior, and a recent survey has shown that 30% of the patients are suffering from borderline personality disorder. The majority of patients (70% to 75%) with borderline personality disorder have a history of deliberate self-harm and 10% have a lifetime risk to die by suicide. The DiaS trial is comparing dialectical behavior therapy with collaborative assessment and management of suicidality-informed supportive psychotherapy, for the risk of repetition of deliberate self-harm in patients with a recent suicide attempt and personality traits within the spectrum of borderline personality disorder. Both treatments have previously shown effects in this group of patients on suicide ideation and self-harm compared with treatment as usual.

**Methods/Design:**

The trial is designed as a single-center, two-armed, parallel-group observer-blinded randomized clinical superiority trial. We will recruit 160 participants with a recent suicide attempt and at least two traits of the borderline personality disorder from the Centre of Excellence in Suicide Prevention, Capital Region of Denmark. Randomization will be performed though a centralized and computer-generated approach that conceals the randomization sequence. The interventions that are offered are a modified version of a dialectical behavior therapy program lasting 16 weeks versus collaborative assessment and management of suicidality-informed supportive psychotherapy, where the duration treatment will vary in accordance with established methods up to 16 weeks. The primary outcome measure is the ratio of deliberate self-harming acts including suicide attempts measured at week 28. Other exploratory outcomes are included such as severity of symptoms, suicide intention and ideation, depression, hopelessness, self-esteem, impulsivity, anger, and duration of respective treatments.

**Trial registration:**

Clinical Trial.gov: NCT01512602.

## Background

In Denmark, the suicide rate is 11.9 per 100,000 people per year according the World Health Organization [1]. Around 8,000 to 10,000 people attempt suicide each year based on information registered in the National Patient Register and the Register of Suicide. Patients who attempted suicide have an increased risk of recurrent suicide attempts and suicidal death [2]. Borderline Personality Disorder (BPD), according to the DSM-IV [3], affects approximately 1% to 2% of the general population, and this patient group represents up to 10% of all psychiatric outpatients and 20% of all inpatients [2,4]. A total of 60% to 70% of the patients with borderline personality disorder will attempt suicide, and the lifetime risk of death by suicide is as much as 10% [5], a risk 50 times higher than in the general population. Approximately 70% to 75% of individuals with BPD, a condition which is associated with challenges in regulating emotions and difficulties in tolerating emotional distress [6], exhibit nonsuicidal self-harm [6].

Dialectical behavior therapy is a manual-based treatment, which was originally developed by the American psychologist Marsha Linehan [6] for the treatment of the core problems of patients with borderline personality disorder, especially concerning emotional regulation, which makes patients particularly prone to self-harming acts. The treatment has shown positive effect in several randomized trials to reduce the high risk of self-injury and suicide attempt in patient with BPD compared with treatment as usual [7-12]. A recent meta-analysis has shown DBT to be the most evidence-based psychotherapeutic treatment for borderline personality disorder and can reduce numbers of self-harming acts and related secondary markers compared with other psychotherapeutic treatments, for example, cognitive behavioral therapy and interpersonal psychotherapy [13]. DBT has also shown effect in treating other dysfunctions such as drug dependence [14] and eating disturbances [15]. A recent systematic review [16] describes seven different studies where the DBT treatment duration varies from 3 to 12 months, and indicates that the most treatment gain is made during the first 4 months of treatment.

The collaborative assessment and management of suicidality (CAMS)-informed supportive psychotherapy was developed by David Jobes and has been somewhat modified in conjunction with Danish colleagues for this study. CAMS is considered both a philosophy of care and a clinical therapeutical framework [17-19]. As a flexible therapeutical framework, CAMS is trans-theoretical and can thereby by used across theoretical orientations, clinical techniques, and professional disciplines. CAMS is designed to specifically target suicidal ideation and behavior as the central clinical focus, independent of diagnosis. Through collaborative assessment and deconstruction of the patient’s suicidality, key problems and goals naturally emerge. Collaborative treatment planning that follows thus creates a problem-focused approach that is designed to reconstruct more viable ways of coping and living. Early studies of CAMS have shown positive effects in reducing suicide ideation in smaller studies with suicidal patients on several parameters [19-23], but there is a need for further research in well-powered clinical trials [24].

The two interventions - DBT versus CAMS - have been chosen for the DiaS trial. They have not previously been compared head to head. This trial will target patients with borderline personality traits and recent suicide attempts.

## Methods/Design

**Figure 1:**
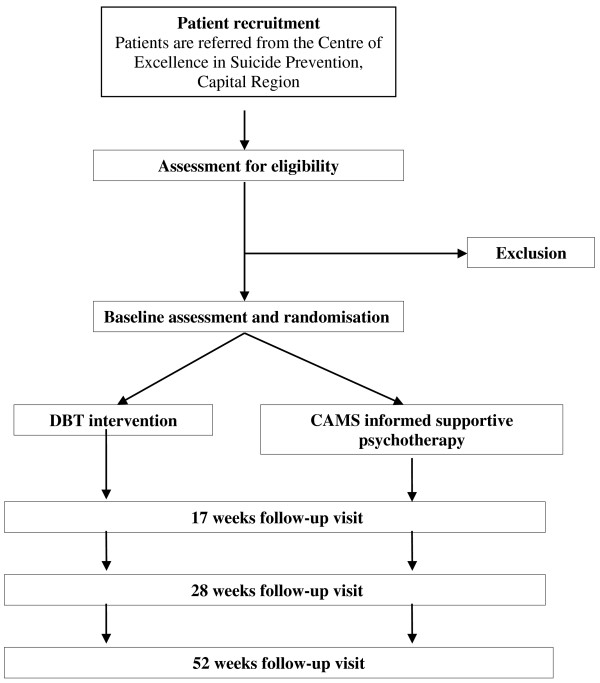
The flowchart of the recruiting and randomization in the DiaS trial.

Our plan is to compare 16 weeks treatment of DBT versus CAMS. The primary outcome will be the ratio of participants with suicide attempts or non-suicidal deliberate self-harming acts (self-reported and registered in medical records) during the treatment period in the treatment period and at follow-up until week 28 after randomization.This trial is designed as a single-center, two-armed, parallel-group, observer-blinded randomized clinical superiority trial (Figure 1).

### Recruitment and criteria for inclusion and exclusion

The patients are recruited through the Centre of Excellence for Suicide Prevention, Capital Region of Denmark. This is a highly specialized outpatient care center treating 450 patients annually and offering short-term supportive psychotherapy and social counseling. The patients are referred from general practitioners and from somatic and psychiatric wards after suicide attempts; patients can also self-refer to the Centre. The patients will be referred to the trial and screened by an assessor. If the patients fulfill the inclusion criteria and none of the exclusion criteria (see list of inclusion and exclusion criteria below), they be will included in the trial and randomized by the first contact therapist in the Centre of Excellence for Suicide Prevention. In the screening interview patients will be subjected to a Mini International Neuropsychiatric Interview (MINI) [25] and a Structured Clinical Interview for DSM-IV Axis II Personality Disorders [26]. Deliberate Self-Harm (DSH) and recent suicide attempts will be measured by using Lifetime Suicide and Self-Injury (L-SASI) [27] and Suicide Attempt and Self-injury Interview (SASII) [28]. The therapist will contact the patients and inform them of which group of the treatment they are to be allocated. The adherence to the treatments in both groups will be measured continuously. Follow-up interviews are performed in week 17, 28, and 52 after inclusion

### Inclusion and exclusion criteria in the DiaS trial

#### Inclusion criteria

1) 18 to 65 years of age.

2) Recent suicide attempt within one month from the inclusion date.

3) At least two of the following criteria for borderline personality disorder (DSM-IV)^a^:

a) Desperate efforts to avoid being betrayed or abandoned in reality or in imagination.

b) A pattern of unstable and intense interpersonal relationships characterized by a swinging between extremes of idealization and devaluation.

c) Identity disturbance: markedly and persistently unstable self-image or self-feeling.

d) Impulsivity in at least 2 areas that are potentially self-harming (for example, sexual misconduct, abuse, consumption, and overeating).

e) Repeated instances of suicidal behavior, gestures or threats or self-harm.

f) Affective instability.

g) Chronic feelings of emptiness.

h) Inappropriate intense anger or difficulty controlling anger.

i) Transient, stress related paranoid ideation, delusions or severe dissociative symptoms.

4) Informed consent.

#### Exclusion criteria

– Severe depression.^b^

– Bipolar disorder.^b^

– Psychosis in schizophrenia spectrum.^b^

– Anorexia nervosa.^b^

– Alcohol or drug addiction.^b^

– Mental retardation.

– Dementia.

– Insufficient ability in speaking and understanding Danish

– Lack of informed consent.

^a^Assessed by SCID-II [26].

^b^Assessed by Mini International Neuropsychiatric Interview (MINI) [25].

All psychiatric departments in the Capital region of Denmark have been informed of the DiaS trial through meetings and leaflets (see inclusion criteria above). Patients with at least two traits from the BPD diagnosis (DSM-IV) [3] and a recent suicide attempt are referred through the Centre of Excellence in Suicide Prevention. The suicide attempt has to be within a month from the first contact with the Centre. The date has been selected to ensure the accuracy of reporting the intent and other important circumstances (feelings, thoughts and behavior prior to, at the time of, and after the suicide attempt). The patients must speak and understand Danish sufficiently to participate in the therapy sessions and the assessment of the trial. The diagnostic criteria for exclusion have been chosen to eliminate potential confounders in the trial and ensure a homogenous group of patients.

### Randomization and blinding

Patients will be randomized into one of two treatment arms. The randomization is stratified by sex and previous number of self-harm acts (one versus multiple). This is done to avoid the risk of patients with multiple self-harm acts being overrepresented in one of the treatment groups. The Copenhagen Trial Unit (CTU) will generate a computer-generated random sequence randomization, using alternating block sizes unknown to the investigators. The trial staff will contact the CTU for information about allocation. The above-mentioned procedure ensures adequate allocation concealment. The patients and therapists will not be blinded. We will use blinded outcome assessors (see outcomes below).

### Dialectical behavior therapy

Dialectical behavior therapy (DBT) consists of four components: 1) individual therapy, 2) skills training in groups, 3) access to telephone contact with therapists, and 4) supervision and consultations for the therapist to prevent burn-out. The first aim of the therapy is organized around a target hierarchy, which consists of 1) eliminating life-threatening behavior including suicide attempts and deliberate self-harm, 2) eliminating treatment interfering behavior such as non-attendance and not doing homework, and 3) ameliorating behaviors leading to a decreased quality of life (axis I disorders, social aspects, and drug dependence). The second aim of the therapy is skill training in groups. Patients are taught four modules of primary skills: 1) mindfulness, 2) emotional regulation, 3) distress tolerance, and 4) interpersonal effectiveness. The individual therapy will focus on the taught skills in the groups. The DBT treatment manual is based on DBT-A by Rathus and Miller [29]. The DBT intervention will consist of 16 weeks treatment defined as 2 hours weekly skill training in groups, 1 hour weekly individual psychotherapy, opportunity of telephone coaching with the therapist, and approximately 1 hour weekly team consultation and supervision.

### Collaborative assessment and management of suicidality-informed supportive psychotherapy

The second intervention consists of supportive psychotherapeutic treatment within the framework of CAMS. CAMS-informed supportive psychotherapy is a psychotherapeutic approach based on principles from the ‘Collaborative assessment and management of suicidality’ [19]. The method typically employs a 45- to 60-minute session once a week in the office of the therapist (in crisis situations the length, frequency, and location of care may vary). CAMS sessions have a specific structure starting in the first 10 to 20 minutes with a collaborative working through of the ‘core assessment’ of the ‘Suicide Status Form’ (SSF), rating on a scale of 1 to 5 the following six suicide-related markers: psychological pain, stress, agitation, hopelessness, self-hate, and overall risk of suicide. In the collaborative exploration of these and additional SSF constructs, the patient and the therapist sit side-by-side. Following the initial assessment, various ‘drivers’ of suicidality are identified and further investigated. Direct drivers are thoughts, feelings, behaviors and interpersonal themes that lead to suicidal thoughts and acts. Indirect drivers are other factors that contribute but do not directly lead to suicidal ideation or feelings, such as: unemployment, depression, substance abuse, post-traumatic stress disorder. A treatment plan is collaboratively formulated that emphasizes a ‘crisis response plan’ to establish outpatient stability. In addition, the CAMS treatment plan further identifies, targets, and treats suicidal drivers with problem-focused interventions. On-going CAMS care involves the development of coping skills for emergencies (for example, the elaboration and use of a crisis response plan) as well as the on-going linking of drivers and suicidality and problem-focused interventions that treat those issues that makes the patient suicidal. This philosophical and clinical approach to treatment is used in subsequent CAMS sessions as the problem-focused treatment to ultimately eliminate suicidal coping as much as possible.

The duration of the CAMS treatment differs depending on the patient’s suicidality. The therapist concludes CAMS treatment after three successive contracts where the patient is assessed to be non-suicidal according to the SSF criteria [17]. In this study, the therapists are supervised one and a half hours every second week.

### Program fidelity

The trial staff consists of four clinical psychologists, two nurses, a social worker, and an occupational therapist. All have had courses and are trained in DBT and CAMS-informed supportive psychotherapy. The training courses offered the therapists were conducted by Alan Fruzzetti, Professor at University of Reno, who trained the staff for 10 days in an intensive DBT training course that consisted of four modules spread over a time period of 8 months and by David Jobes, who held a 2-day course twice on CAMS-informed supportive psychotherapy. The individual sessions with the patients will be videotaped and used to rate the adherences in both interventions. In the DBT group the rating scale to be used will be the DBT-global rating scale by Marsha Linehan (unpublished work). The therapist adherence to CAMS-informed supportive psychotherapy will be assessed with the CAMS rating scale (DA Jobes, unpublished work).

The CAMS intervention will be carried out at the established departments of the Centre of Excellence for Suicide Prevention, Capital Region of Denmark, Mental Health Services, Psychiatric Centre of Copenhagen and Psychiatric Centre of Amager. The DBT intervention will be carried out at the Centre of Excellence for Suicide Prevention, Psychiatric Centre of Copenhagen.

### Assessments

The patients will be subjected to four almost identical assessments. The first baseline assessment will be performed in continuity with the assessment and validation of the inclusion and exclusion criteria. Also the strata used for the randomization will be obtained in the first interview with the patient. The second assessment will occur after treatment has ended in week 17 after inclusion in the trial. The third and fourth assessment will occur at a follow-up interview after week 28 and week 52 after inclusion.

### Outcomes

#### Primary outcome

The primary outcome will be measured as the ratio of deliberate self-harm before and after treatment (week 0 compared with week 28). The Suicide attempt and Self-Injury Interview (SASII) [28] will be used to collect these data and direct questions in the follow-up interview regarding self-harming behavior after inclusion in the trial. We will also obtain information concerning numbers of deliberate self-harm and suicide attempts through medical records.

#### Explorative outcomes

In our trial we have chosen a number of exploratory outcomes, which are not based on power calculations. These include the severity of BPD symptoms, suicide intention and ideation, depression, hopelessness, self-esteem, impulsivity, and anger. We will also examine between-group differences in ‘dosage’ and durations of the respective treatments.

#### Borderline personality disorder symptoms

The severity of features regarding borderline personality symptoms is measured by the Zanarinis Rating Scale for BPD. It is a nine-item, five-point scale (0 to 4, 0 = no symptoms and 4 = severe symptoms) validated, clinician-based diagnostic interview (DSM-IV) [30]. The categories of symptoms are affective, cognitive, impulsivity, and interpersonal symptoms.

#### Suicide intention and ideation

Suicide intention is measured by Beck’s Suicide Intent Scale (SIS) [31,32]. It consists of 20 items, where the different circumstances and practical preparations are investigated.

The Beck Suicide Ideation Scale (BSS) is a 19-item self-report questionnaire measuring suicidal thinking [32]. Items are scored on a Likert scale ranging from 0 to 2, with a higher score indicating more severe suicidal ideation.

#### Depression

Depressive symptoms will be assessed by Hamilton Depression Rating Scale (HDRS) 17-items [33]. The patients are also subjects to Beck’s Depression Inventory (BDI) [34,35], a 21-item self-report inventory of the depressive symptomatology measuring depression. The BDI is the most widely used research instruments and the items are rated on a Likert scale from 0 to 3, where higher scores indicate a more severe level of depressive symptoms.

#### Hopelessness, self-esteem, impulsivity, anger

The Becks Hopelessness Scale (BHS) consists of 20 true-false items pertaining to future outlook [36]. Rosenberg’s Self- Esteem scale (RSE) is used to assess the self-esteem. It consists of 10 items and is a Likert scale with answers using a four-point scale -from strongly agree to strongly disagree [37].

Barratts Impulsivity Scale 11 (BIS-11) is a 30-item questionnaire measuring impulsive personality traits [38]. It yields a total score, three second-order factors, and six first-order factors [39]. To measure anger, we will use State Trait Anger Scale (STAS), developed by Charles Spielberger [40]. It consists of 20 items in a self-report questionnaire and is rated on a Likert scale with answers using a four-point scale -from never to always.

### Sample size and statistical analyses

Based on previous estimations, we expect the risk of renewed self-harming acts within their first year to be 50 percent in the CAMS treatment group [6]. We expect the DBT to be able to reduce this risk to 25 percent, a relative risk reduction of 50%. With a power of 90% and a type one error probability associated with this test of 0.05, we plan to randomize 154 patients, with about 77 patients in each intervention group. We will handle missing data using logistic regression with multiple imputations as described below.

The two group baseline characteristics will be compared using Student’s *t*-test for independent samples or a chi-square test for binary variables.

The primary analysis will be based on the intention-to-treat principle; that is, data from all patients will be included in the treatment group to which they were allocated regardless of degree of compliance.

Binary outcomes will be analyzed using logistic regression and continuous outcomes will be analyzed using linear regression with baseline variables as sex and previous deliberate self-harm as a covariates.

If more than 5% of the primary outcome is missing at follow-up, we will use multiple imputations (SPSS version 19.0). If this is the case, the multiple imputations will be considered the primary result. For multiple imputations, we will use a linear regression model with 100 imputations and 20 iterations. The pooled estimates from the imputations will subsequently be used for our analysis.

We will use two-tailed tests for statistical significance with alpha set at *P* <0.05.

### Ethical considerations

The trial protocol is approved by the regional ethics committee in the Capital Region of Denmark under file number H-1-2011-042. The Danish Data Protection Agency has approved the management of data in the trial under the file number 2007-58-0015. Finally, the trial is registered under ClinicalTrial.gov as NCT01512602. In accordance with the CONSORT guidelines [41], we will publish negative, neutral and positive findings in the trial.

We have decided not to have a control group without any intervention, because of the ethical aspects when treating suicidal and vulnerable patients.

The patients will be informed about the trial with both verbally and with written information before signing the written consent. It will be stressed that participation in the trial is voluntary. Participation and the written consent can be withdrawn at any time in the trial, and this can be done with no consequence for future treatment possibilities.

## Discussion

The DiaS trial is designed as a pragmatic trial, with the intention of intervening in the exiting clinical outpatient setting, so one could argue the patient population is selected beforehand. The Excellence Centre for Suicide Prevention has their exclusion criteria before offering treatment as mentioned before and can exclude patients for whom treatment can appear too demanding. The chosen target group includes patients with emotional dysregulation leading to suicide attempts. This patient group is over-represented in the Centre of Excellence in Suicide Prevention. The chosen experimental DBT treatment is based on a modified DBT manual but where all the skills taught are maintained. The DBT treatment period of 16 weeks is shorter than in the majority of other randomized clinical trials performed [16]. The considerations of these modulations are the excepted occurrence of the lesser chronicity and severity in BPD symptoms in the target group of patients. The patients only have to fulfill two out of nine BPD criteria according to the DSM-IV. Regarding the CAMS-informed supportive psychotherapy given to the control group, this treatment has been an obvious choice as the majority of therapist in the Excellence Centre for Suicide Prevention use this treatment. This treatment group can be considered as an optimized ‘treatment-as-usual’ control group, where the therapists are trained and supervised, and treatment adherence is rated.

The two different intervention groups will receive different amounts of time of individual psychotherapy, and the CAMS group is not offered skill training in groups. We consider the different amounts of time in the two groups as inevitable because of the different basic construction of the two psychotherapeutic treatments. In the CAMS- intervention group the therapist has to finish the treatment when achieving three consecutive non-suicidal scores on the SSF. A strength of this trial is the centralized randomization [42], which reduces the risk of selection bias. We will perform blinded outcome assessment to reduce bias [43]. We will use intention-to-treat analysis and multiple imputations to reduce the bias introduced by missing data in our analysis [43].

A related post-hoc consideration is the potential ‘cost-effectiveness’ of the respective treatments in relation to frequencies of hospitalizations, emergency department visits, and primary care visits. The cost-effectiveness of clinical care for high-risk patients was previously examined in a retrospective study of CAMS versus treatment-as-usual with suicidal outpatients [44] and is a topic of increasing importance within contemporary mental health care around the world.

The DiaS trial is a single center trial and can therefore be considered as a pilot trial for multicenter trials in the future. In order to obtain external validity, we need multicenter trials. The DiaS trial has a 52-week follow-up period to investigate deliberate self-harm and suicide attempts after treatment. This knowledge can be useful in the design of trials, in regard to treatment duration or to booster treatment sessions. Moreover we need trials showing comparable effects both during the intervention period and the post-intervention period.

## Trial status

The status of the trial is recruitment of patients, which began in January 2012 and is expected to finish in January 2014. We must recruit 154 participants, and we find this goal realistic because it is estimated that approximately 30% of the patients in the target group at the Excellence Centre for Suicidal Prevention will fulfill the inclusion criteria for the DiaS trial.

## Abbreviations

BDI: Beck’s depression inventory; BPD: borderline personality disorder; BHS: Beck’s hopelessness scale; BIS-11: Barratt’s impulsivity scale 11; BSS: Beck’s suicide ideation scale; CAMS: collaborative assessment and management of suicidality; CTU: Copenhagen trial unit; DBT: dialectical behavior therapy; DBT-A: dialectical behavior therapy- adolescent; DSH: self liberate self-harm; DSM-IV: Diagnostic and Statistical Manual of Mental Disorders-4th edition; L-SASI: lifetime suicide and self-injury interview; HDRS-17: Hamilton depression rating scale-17; RSE: Rosenberg’s self-esteem scale; SASII: suicide and self-injury interview; SIS: Beck’s suicide intent scale; SSF: suicide status form; SPSS: Statistical Package for the Social Sciences.

## Competing interests

The authors declare that they have no competing interests.

## Authors’ contributions

KTA was responsible for the study conception and the design of the trial protocol, and writing the manuscript. MN was responsible for study conception and the design of the trial protocol, writing the manuscript and critical revision of the work. JK participated in the design of the trial, writing the manuscript, and critical revision of the work. BR participated in writing the manuscript and critical revision of the work. CG participated in the design of the trial, writing the manuscript and critical revision of the work. DJ participated in writing the manuscript and critical revision of the work. All authors read and approved the final manuscript.
